# Highlight: Striking Discoveries in Rattlesnake Venom Evolution

**DOI:** 10.1093/gbe/evae137

**Published:** 2024-07-12

**Authors:** Casey McGrath

Snake venom—an intricate blend of toxins, proteins, and peptides—exhibits astonishing variation in composition, even among closely related populations and species. Understanding this diversity is crucial for better understanding venom evolution, as well as improving antivenom development. However, until now, the genomic changes driving these differences in venom composition have remained elusive. According to Siddharth Gopalan, a doctoral student, and Todd Castoe, his advisor at the University of Texas at Arlington, “Little was known about the molecular underpinnings that generate the extensive variation observed in venom and venom gene expression, including how variation in different regulatory components (i.e., promoters, enhancers, transcription factors) manifests in venom compositional differences at shallow evolutionary time scales.” To shed light on these processes, the researchers embarked on an in-depth study of the prairie rattlesnake *Crotalus viridis* and its close relatives. Their findings, published in a new article in *Genome Biology and Evolution*, uncovered a diverse array of genomic mechanisms that shape the venom profiles of closely related rattlesnake species (Gopalan et al. 2024).

Gopalan, Castoe, and their colleagues integrated a suite of omics technologies, including transcriptomics, proteomics, whole genome resequencing, and ATAC-seq (a sequencing-based assay for measuring chromatin accessibility), to explore correlations between venom phenotypes and changes in the regulation of venom genes. The study included *C. viridis* (Fig. 1) and its close relatives, *Crotalus oreganus concolor*, *Crotalus oreganus lutosus*, and *Crotalus cerberus*. The venom profiles—including both the composition and expression levels of venom genes—differ significantly between southern and northern populations of *C. viridis* and among the four species of rattlesnake, providing a useful model system for studying the functional genomic underpinnings of venom phenotypic variation. In addition, the relatively young divergence times among members of this clade allowed the researchers to link variation in venom phenotypes to specific changes in the gene regulatory network.

**Fig. 1.**
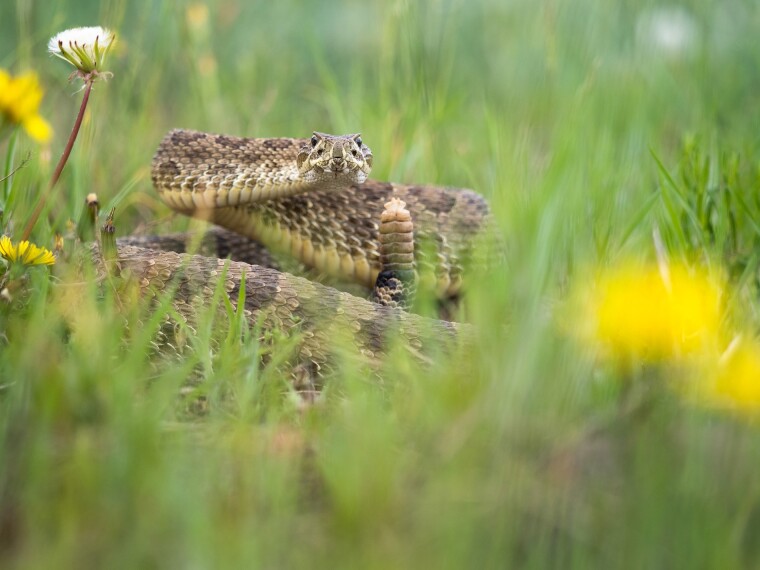
Photograph of the prairie rattlesnake *C. viridis*. Credit: Yannick Francioli.

The study revealed substantial diversity in the expression of numerous venom genes and proteins across individual rattlesnakes, with particularly high variation in the expression of myotoxins, snake venom metalloproteinases, members of the phospholipase A_2_ family, and snake venom serine proteases. These proteins were often coexpressed with venom-associated transcription factors, chromatin regulators, and other transcription factors related to the extracellular signal-related kinase (ERK) and unfolded protein response (UPR) pathways, which are hypothesized to coordinate venom expression. Notably, each of the four rattlesnake species exhibited distinct coexpression networks, suggesting that venom variation may be partly driven by differences in the expression of trans-regulatory factors, especially across species.

The authors also investigated changes in cis-regulatory elements associated with venom loci, including both promoters (sequences located just upstream of genes that drive transcription) and enhancers (sequences that may activate transcription over longer distances). Interestingly, enhancers consistently showed greater variation than promoters in terms of both chromatin accessibility and transcription factor binding, while promoters displayed greater nucleotide diversity than enhancers. Moreover, nearly half of the sequence variants observed in transcription factor binding sites were unique to a single sampled individual, highlighting this as a key mechanism driving variation in venom expression across populations and species.

According to the authors, these results suggest that “even at shallow levels of divergence, a diversity of regulatory mechanisms may shape phenotypic variation, with distinct genomic mechanisms dominating the modulation of gene expression for particular genes and gene families.” These mechanisms may include changes in cis-regulatory elements that modify transcription factor binding sites, variable transcription factor expression, and changes in chromatin accessibility. “There isn’t just one consistent way or mechanism by which venom diversity arises,” continue Gopalan and Castoe. “Instead, a wide range of distinct molecular mechanisms have been leveraged by evolution to modulate gene expression to produce incredibly variable and distinct venom profiles within and across rattlesnake species.” The authors further note that there may be multiple underlying forces driving this diversity in venom profiles, including balancing selection consistent with predator–prey arms races, geographically distinct prey populations, and regional abiotic factors such as temperature.

One caveat of the study, as noted by the authors, is the lack of information on the role of nonprotein-coding RNAs in the evolution of venom gene regulation. The researchers observed multiple venom genes with lower-than-expected protein abundances given their transcript expression levels, consistent with a potential posttranscriptional regulatory mechanism that may involve microRNAs and other noncoding RNAs. This represents an important area of investigation for future studies. In addition, the research team plans to “integrate data from single-cell experiments, alongside evolutionary variation, to further test the contributions of specific regulatory elements to phenotypic variation and more powerfully test hypotheses for gene regulatory network function and genotype-phenotype relationships.”

In addition to shedding light on the mechanisms of snake venom evolution, this research has important implications for the treatment of snakebites, which the World Health Organization has identified as a key priority. The North American rattlesnakes in this study exhibit incredible diversity in venom composition, which the authors note is also linked to extensive variation in biological activity and medical outcomes in the context of snakebite: “Understanding this variation and the mechanisms that generate it are of major relevance to snakebite treatment and how that treatment may vary based on variation in venom activity observed across populations and species.”

***Want to learn more?*** Check out these other articles on snake venom evolution recently published in *Genome Biology and Evolution*:

- Single-Cell Heterogeneity in Snake Venom Expression Is Hardwired by Co-Option of Regulators from Progressively Activated Pathways (Westfall et al. 2023)
- De Novo Genome Assembly Highlights the Role of Lineage-Specific Gene Duplications in the Evolution of Venom in Fea’s Viper (*Azemiops feae*) (Myers et al. 2022)

## References

[evae137-B1] Gopalan SS, Perry BW, Francioli YZ, Schield DR, Guss HD, Bernstein JM, Ballard K, Smith CF, Saviola AJ, Adams RH, et al Diverse gene regulatory mechanisms alter rattlesnake venom gene expression at fine evolutionary scales. Genome Biol Evol. 2024:16:evae110. 10.1093/gbe/evae110. 38753011 PMC11243404

[evae137-B2] Myers EA, Strickland JL, Rautsaw RM, Mason AJ, Schramer TD, Nystrom GS, Hogan MP, Yooseph S, Rokyta DR, Parkinson CL. De novo genome assembly highlights the role of lineage-specific gene duplications in the evolution of venom in Fea’s viper (*Azemiops feae*). Genome Biol Evol. 2022:14(7):evac082. 10.1093/gbe/evac082. 35670514 PMC9256536

[evae137-B3] Westfall AK, Gopalan SS, Perry BW, Adams RH, Saviola AJ, Mackessy SP, Castoe TA. Single-cell heterogeneity in snake venom expression is hardwired by co-option of regulators from progressively activated pathways. Genome Biol Evol. 2023:15(6):evad109. 10.1093/gbe/evad109. 37311204 PMC10289209

